# Study of the collagen type VI alpha 3 (*COL6A3*) gene in Parkinson’s disease

**DOI:** 10.1186/s12883-021-02215-7

**Published:** 2021-05-08

**Authors:** Chong-Yao Jin, Ran Zheng, Zhi-Hao Lin, Nai-Jia Xue, Ying Chen, Ting Gao, Yi-Qun Yan, Yi Fang, Ya-Ping Yan, Xin-Zhen Yin, Jun Tian, Jia-Li Pu, Bao-Rong Zhang

**Affiliations:** Department of Neurology, Second Affiliated Hospital, College of Medicine, Zhejiang University, Hangzhou, Zhejiang 310009 People’s Republic of China

**Keywords:** Parkinson’s disease, Dystonia, Gene, *COL6A3*

## Abstract

**Background:**

To date, the genetic contribution to Parkinson’s disease (PD) remains unclear. Mutations in the collagen type VI alpha 3 (*COL6A3*) gene were recently identified as a cause of isolated dystonia. Since PD and dystonia are closely related disorders with shared clinical and genetic characteristics, we explored the association between *COL6A3* and PD in a Chinese cohort.

**Methods:**

We performed genetic screening of *COL6A3* in a Chinese cohort of 173 patients with sporadic PD and 200 healthy controls. We identified variants that are likely to have pathogenic effects based on: 1) a minor allele frequency of < 0.01; and 2) the variant being recognized as deleterious by at least 15 different in silico predicting tools. Finally, we tested the aggregate burden of *COL6A3* on PD via SKAT-O analysis.

**Results:**

First, we found compound heterozygous *COL6A3* gene mutations in one early-onset PD patients. Then, we explored whether *COL6A3* variants contributed to increased risk of developing PD in a Chinese population. We detected 21 rare non-synonymous variants. Pathogenicity predictions identified 7 novel non-synonymous variants as likely to be pathogenic. SKAT-O analysis further revealed that an aggregate burden of variants in *COL6A3* contributes to PD (*p* = 0.038).

**Conclusion:**

An increased aggregate burden of the *COL6A3* gene was detected in patients with PD.

## Background

Parkinson’s disease (PD) is the second most common neurodegenerative disease in the world, affecting approximately 2–3% of the population ≥ 65 years of age [1, 2]. The disease is characterized by multiple symptoms, grouped as motor symptoms such as bradykinesia, resting tremor and rigidity [3], and non-motor symptoms such as depression, apathy and sleep disorders [4]. The precise cause of PD is currently unknown, although scientists generally believe that it is a result of both genetic and environmental factors.

The collagen type VI alpha 3 (*COL6A3*) gene, encoding the collagen alpha-3(VI) chain, is recognized as being associated with muscular dystrophy [5]. Recently, Zech et al. found that loss-of-function mutations of *COL6A3* cause autosomal recessive isolated dystonia [6], a movement disorder characterized by intermittent muscle contractions [7, 8]. Dystonia occurs either as an isolated condition or accompanied by other disorders such as Parkinsonism, and is closely linked to PD. Dystonia is seen in over 30% of patients with PD and often even before the onset of Parkinsonism [9]. Dystonia is common in PD patients whose age of onset is < 40, and it most commonly affects the lower limbs [10, 11].

Several previous studies have found that patients carrying mutations in dystonia-related genes, such as *GCH1* and *TH*, displayed features of Parkinsonism in addition to dystonia [12, 13]. Even in patients with pure Parkinsonism (without dystonia), the frequency of rare *GCH1* variants were higher than in controls, indicating that variants in *GCH1* may be associated with a higher risk of PD. [14] Extending current understandings of the *COL6A3* gene, we report here compound heterozygous mutations in the *COL6A3* gene in an early-onset PD patient. To further investigate the relationship between *COL6A3* and PD, we screened for *COL6A3* mutations in a Chinese cohort.

## Methods and materials

### Subjects

A total of 173 cases of sporadic PD (diagnosed by a specialist in movement disorders following the MDS diagnostic criteria [3]) were recruited from the Second Affiliated Hospital of Medicine College, Zhejiang University, between January 2016 and June 2019. Of these, 81 were defined as early onset PD (EOPD) with age at onset ≤50 years old. The remaining 92 were late onset PD (LOPD) with age at onset > 50 years old A gene sequencing panels test of PD was performed in all patients and no pathogenic variants in established Mendelian Parkinson’s disease genes such as *SNCA, LRRK2, VPS35, PARK2/parkin, PARK7/DJ-1, or PINK1* were found. The index patient was a 43 years old male with no remarkable medical history or family history from Chinese Han population. In addition, 200 demographically-matched healthy controls were included.

### Sample preparation and sequencing

Genomic DNA of the patients and controls was isolated from peripheral leukocytes using standard protocols. Whole sequences of the selected genes were captured with a SureSelect Human All Exon 50 Mb kit (Agilent Technologies). Sequencing was conducted as 150 bp paired-end runs on an Illumina Nova Seq 5000 system to a 300-fold depth of coverage. AfterQC [15] was used to processes the raw data, including filtering out and trimming bad reads. Then, sequence reads were mapped to the human genome assembly GRCh37/hg19 using Burrows–Wheeler Aligner 16. ANNOVAR software was used to annotate the variants [16].

### Criteria for pathogenicity of rare variants

To determine the deleterious variants of the *COL6A3* gene, we filtered the variants found in our cohort using the following algorithm: 1) identify rare non-synonymous variants with a minor allele frequency of < 1% or “not available” in the Genome Aggregation Database East Asia [17] or the 1000 Genomes Project [18]. Variants in the exon region were selected for further evaluation since they are more likely to have a negative effect on protein structure and function. 2) Use an integrated genetic and clinical database, the VarCards [19], for preliminary evaluation of the deleterious effect of the variants. Those variants predicted to be deleterious by at least 15 tools were considered potentially pathogenic. 3) Screen the variants in the control population.

Amino acid conservation analysis using multiple sequence alignment of *COL6A3* protein sequences from different species was performed by ClustalX [20].

The 3D protein structures of the wild-type and variant proteins were predicted using Phyre2 [21] and visualized by PyMOL (The PyMOL Molecular Graphics System v2.0, Schrödinger, LLC). The effects of missense variants on protein structure were evaluated by Missense3D [22].

### Statistical analysis

Before genetic analysis was performed, we carried out a Hardy–Weinberg Equilibrium (HWE) test via chi-squared test in the control group [23].

The sequence kernel association test optimal (SKAT-O [24],) was implemented in R (version3.6.2, The R Foundation) using SKAT v2.0 to determine the difference between PD cases and controls in aggregate burden of rare *COL6A3* gene variants. Gender was adjusted as a covariate. Power calculations were performed using functions in the SKAT-O R packages with disease prevalence of PD set as 1.7%, significant lever set as 0.05 and causal percent of the variants set as 10,30 and 50% respectively.

## Results

### General information

**Fig. 1:**
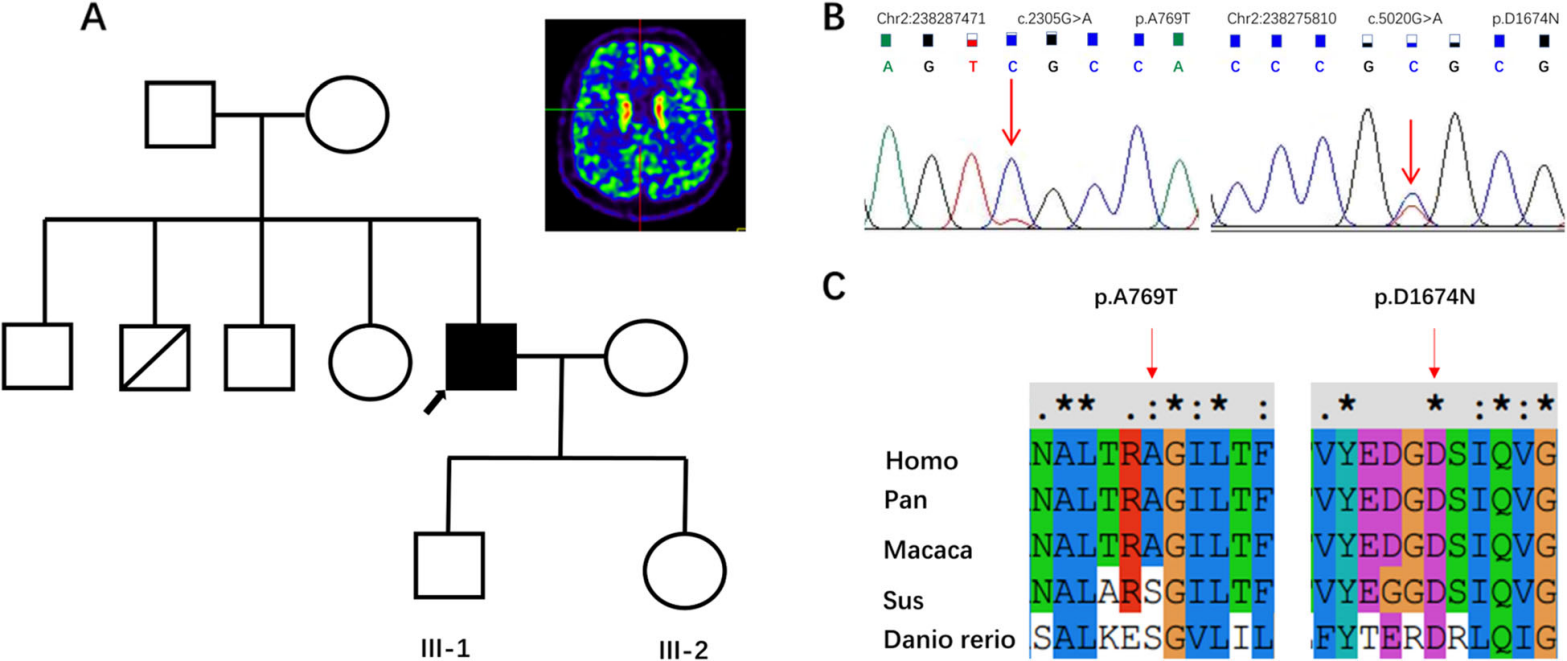
**a**: Pedigrees and DAT-PET scan images of the index patient. II-2 died of liver cancer **b**: sanger sequencing result. **c**: multiple sequence alignment conducted by ClustalX. Asterisk (*) indicates positions that have a single, fully conserved residue. Colon (:) indicates conservation between groups of strongly similar properties. Period (.) indicates conservation between groups of weakly similar properties

Our index patient was a 43-year-old male with no remarkable past medical history. He developed a mild gait difficulty 4 years ago. His symptoms worsened over time and he gradually experienced muscle rigidity and clumsiness. Physical examination showed bilateral rigidity and bradykinesia without resting tremor or dystonia. Dopamine transporter positron emission tomography (DAT-PET) revealed reduced tracer uptake bilaterally, mainly in the putamen, indicating nigrostriatal dopaminergic denervation. He responded well to levodopa therapy (300 mg/day). Whole-exome sequencing didn’t found any mutation in known PD genes, however, revealed two heterozygous recessive variants in the *COL6A3* gene (p.A769T and p.D1674N). The p.A769T variant was reported on ClinVar with an interpretation of uncertain significance or likely benign, while the other variant p.D1674N was not previously reported on ClinVar. His pedigree chart and DAT-PET scan result are shown in Fig. 1.

General information about our cohort were presented in Table 1. Among the PD patients, 12 patients were recorded with dystonia and the prevalence was higher in EOPD group than LOPD group.

**Table 1:** summary of basic information of PD and Control

	PD			Control	*P*
	All	EOPD	LOPD		
Number	173	81	92	200	.
Male	93 (53.8%)	42 (51.9%)	51 (55.4%)	102 (51%)	0.61
AAO ± SD	52.22 ± 10.03	44.32 ± 5.46	59.17 ± 7.72	.	.
Disease duration	3.35 ± 3.09	3.81 ± 3.82	2.93 ± 2.20	.	
Dystonia feature	12 (6.9%)	8 (9.8%)	4 (4.3%)		

EOPD is defined in this study as PD patients whose age of onset is ≤50, while LOPD is defined as patients whose age of onset is > 50. *P* value refer to comparison between control and all PD patients. *AAO* Age at onset, *SD* Standard deviation

### Variant analysis

Most genotype frequencies in the control group were in agreement with the HWE principle (*p* > 0.05), indicating our data are free from sample level substructure and genotyping error.

According to our filtering procedure, 21 rare non-synonymous variants were selected (data not shown). In silico predicting tools further identified seven patients with variants of *COL6A3* likely to have a pathogenic effect (Table 2). Of these patients, four were in the EOPD group and the other three in the LOPD group. Only two patients with the variants p.A1031T and p.R1656Q presented with foot dystonia. Detailed clinical features of the other patients are shown in Table 3.

**Table 2:** Information of probable pathogenic variants identified in our cohort

RefSeq	Variants	Exon	Nucleotide change	Protein change	Population MAF (East Asian)		Frequency in our cohort			In silico prediction		
					gnomAD	1000 G	PD	control	P value^#^	CADD	SIFT	Deleterious vs all algorithms^a^
NM_057166.4	rs545819982	36	c.5692G > A	p.G1898R	0	0.0002	1/346	0/400	0.038	20.5	0	18:23
	rs369317566	31	c.5204G > A	p.R1735Q	0	.	1/346	0/400		24.3	0.365	16:23
	rs370862741	29	c.5095C > T	p.R1699C	0.0001	0	1/346	0/400		24.4	0.055	19:23
	rs779508804	27	c.4967G > A	p.R1656Q	0.0001	.	1/346	2/400		24.5	0.053	16:23
	rs770876436	12	c.3707 T > C	p.F1236S	0.0003	.	1/346	0/400		24.6	0.002	16:23
	rs114322958	11	c.3091G > A	p.A1031T	0.0063	0.0008	1/346	9/400		25.5	0.005	20:24
	rs115765346	10	c.2783G > A	p.R928H	0.0001	0	1/346	2/400		27.3	0.002	22:23

^a^algorithms or tools include PolyPhen2_HDIVPolyPhen2_HVAR, LRT, MutationTaster, MutationAssessor, FATHMM, PROVEAN, MetaSVM, MetaLR, VEST3, M-CAP, CADD, GERP++, DANN, fathmm-MKL, Eigen, GenoCanyon, fitCons, PhyloP, PhastCons, SiPhy and REVEL

^#^*P* value calculated via SKAT-O

**Table 3:** Clinical manifestation of patients with *COL6A3* variants that are likely pathogenic

Gender	Age of onset	Amino Acid Change	Disease duration	Parkinsonian features	Dystonia feature	Levodopa response
Female	59	R928H	5	bradykinesia and rigidity, Loss of smell, sleep disturbance and constipation	none	good
Female	57	A1031T	2	right lower limb tremor and slightly impaired gait	right foot dystonia	not taken
Male	47	F1236S	2	right limbs resting tremor	none	not taken
Female	47	R1656Q	4	tremor, rigidity and bradykinesia, mainly in the right limb; constipation	right foot dystonia	good
Male	49	R1699C	3	right limb resting tremor and bradykinesia; loss of smell and fatigue	none	good
Male	45	R1735Q	3	bradykinesia and impaired gait, pain	none	good
Male	56	G1898R	7	hallucinations, dementia, insomnia and slurred speech, bradykinesia and rigidity	none	not good

**Fig. 2:**
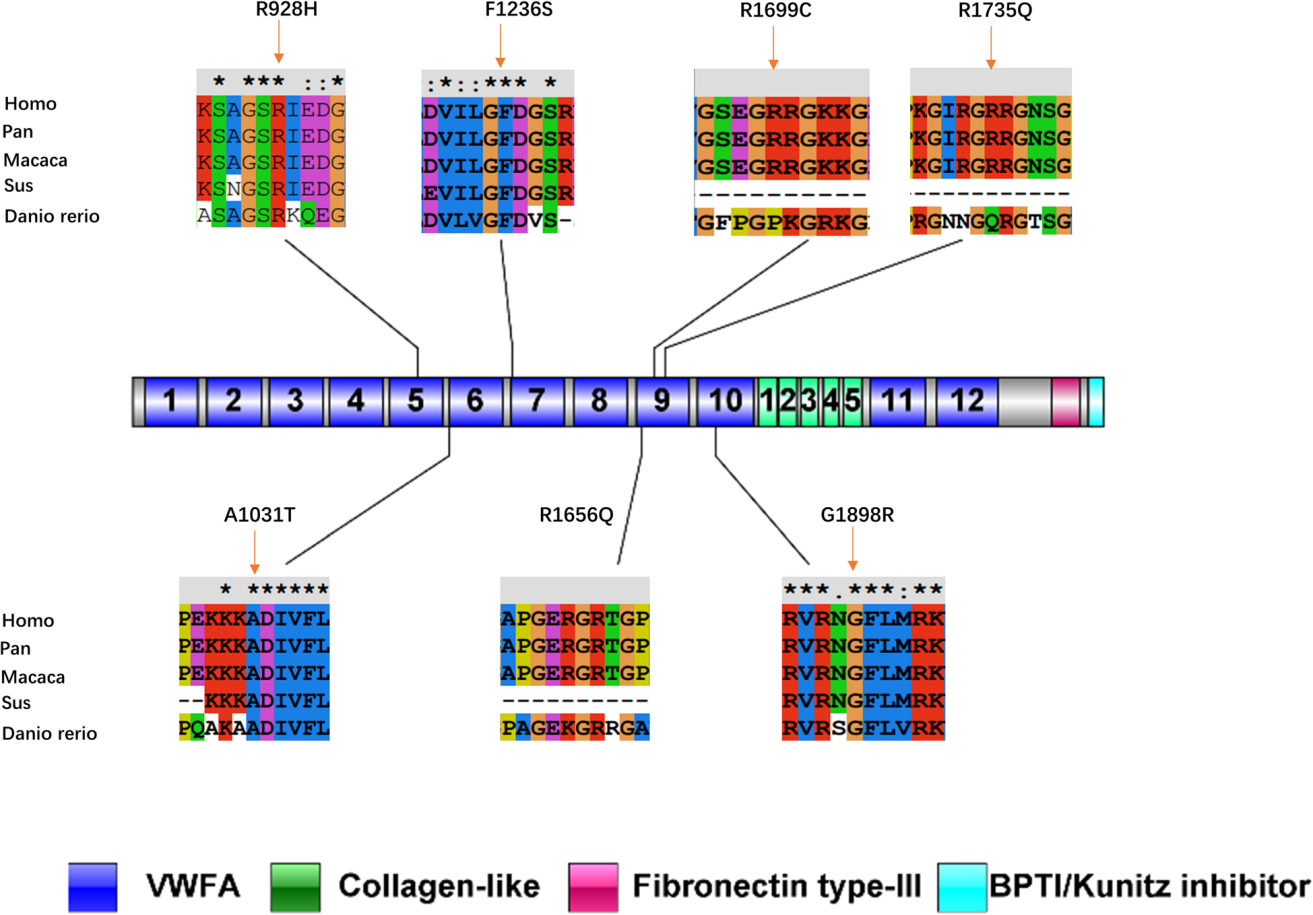
Schematic of gene coding regions and locations of novel variants detected in this cohort and its multiple sequence alignment carried out by the program ClustalX. Asterisk (*) indicates positions that have a single, fully conserved residue. Colon (:) indicates conservation between groups of strongly similar properties. Period (.) indicates conservation between groups of weakly similar properties

**Fig. 3:**
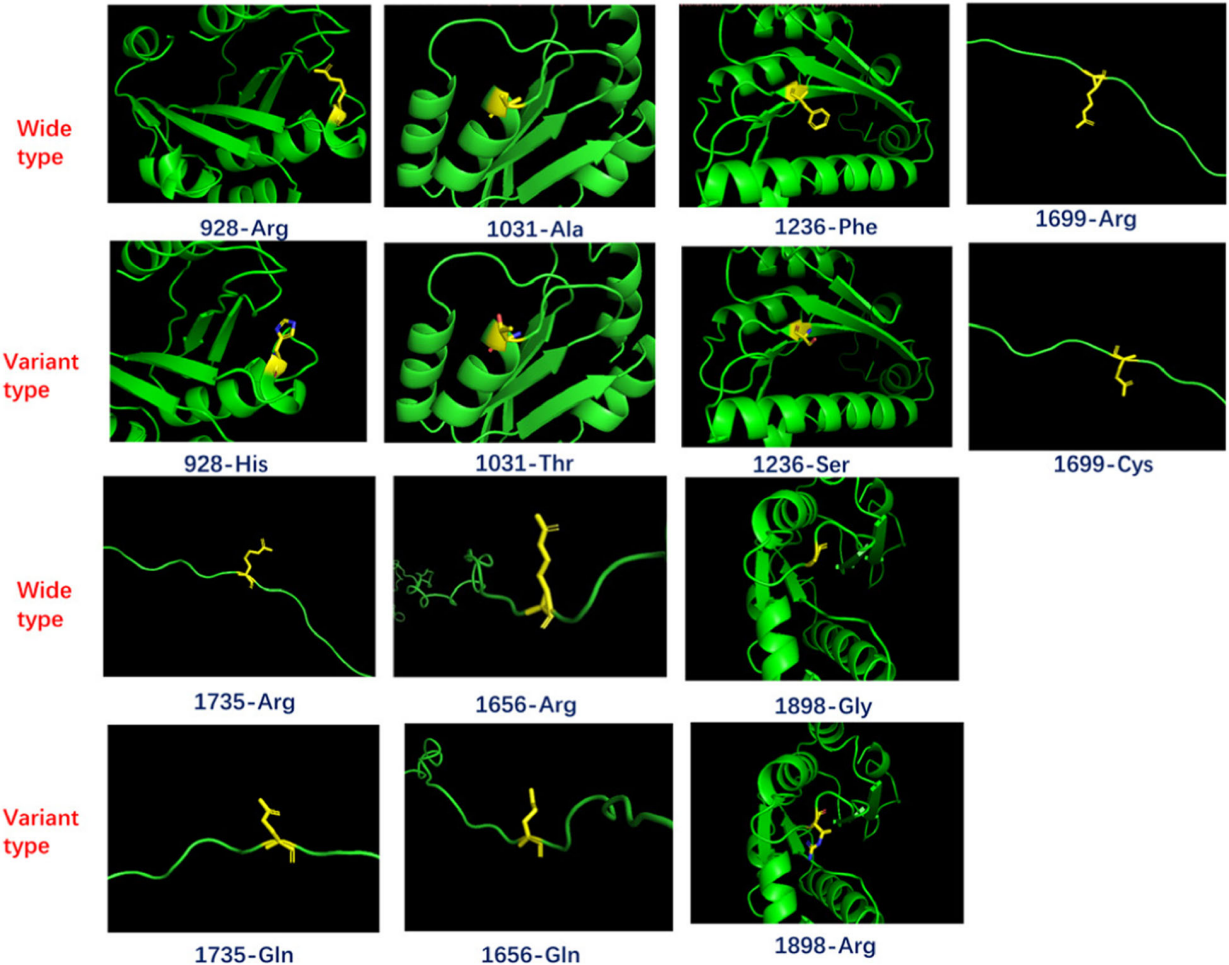
The 3-D structures of wild type and variant-type proteins. Protein models were shown in secondary structures

Multiple sequence alignment by ClustalX showed that four variants were fully conserved (p.R928H, p.A1031T, p.G1898R and p.F1236S), indicating variants in these loci may have an effect on protein function (Fig. 2). However, 3D structure prediction of all the variants and the wild type detected no structural alterations (Fig. 3).

Of the seven variants we identified, two were previously reported on Clinvar. These two, rs114322958 and rs545819982, were recorded as benign and uncertain significance respectively for Bethlem myopathy 1.

SKAT-O analysis showed significant associations for the *COL6A3* gene between PD patients and controls considering the 7 likely damaging variant we identified above after adjusting for gender. (*P* = 0.038). Statistical powers were 13.2, 19.2 and 39.5% respectively when assuming the casual percent to be 10, 30 and 50%.

## Discussion

The *COL6A3* gene, encodes a component of type VI collagen, which is a flexible protein in the extracellular space [25]. Previous studies [26] have shown that mutation in *COL6A* genes (*COL6A 1–3*) is associated with Bethlem myopathy and Ullrich congenital muscular dystrophy. Now it is believed that these two diseases may be at one end of a phenotypic spectrum, mainly manifesting as muscle weakness and joint contractures [27].

Using whole-exome sequencing, Zech et al. [6] recently reported recessive mutations in the *COL6A3* gene in association with early-onset isolated dystonia. Clinical symptoms varied from focal dystonia, mild cervical dystonia, mild segmental dystonia and mild generalized dystonia to severe generalized dystonia. A previous study by Zech et al. [28] detected five variants in five early-onset isolated dystonia patients, and found that at least one of the homozygous variants is located in exon 41 or 42. Furthermore, experiments in Zebrafish embryos and the mouse brain showed the exon skipping mutation in exon 41 resulted in the development of segmental dystonia, without any muscular disease. Thus, they concluded that variants in *COL6A3* may cause dystonia by affecting the extracellular matrix in the central nervous system, and that exons 41 and 42 are hotspots for mutation [6]. However, the role of *COL6A3* mutation in isolated dystonia had been challenged by Lohmann et al. [27], who screened 955 patients with combined or isolated dystonia. They only identified one biallelic mutation in a patient with Parkinsonism and dystonia. This patient also carried homozygous mutations in the *PINK1* gene, which are considered to be associated with early-onset Parkinsonism. Panda et al. also reported an early-onset isolated dystonia case with two pathogenic compound heterozygous loss-of-function mutations in exons 10 and 12 of *COL6A3* [29].

Dystonia can be seen in both early- and late-onset PD patients, and can occur precede or after parkinsonism [30]. Two of the seven patients we identified carrying these possible deleterious variants presented with dystonia feature. We assume it may be due to the relatively short disease duration (mean 3.7 years).

In the present study, we reported two recessive mutations (p.A769T and p.D1674N) in the *COL6A3* gene in our index PD patient. The variants are located in exons 6 and 8, in contrast to the previous study. However, since the patient currently shows no symptoms of dystonia, we assume variants in some region of *COL6A3* may have an association with PD. As a result, we identified seven likely pathogenic variants in *COL6A3*, but none of them were located in exon 41 or 42 as previously reported. In addition, two patients who carried the variants p.A1031T and p.R1656Q presented with dystonia. SKAT-O analysis showed significant aggregate burden between patients and controls, indicating that variants in the *COL6A3* gene may increase the genetic burden in PD.

Conventional single-variant test may not be appropriate in our cohort because of small sample size and variants’ low frequencies. Thus we adapted the SKAT-O to investigate the associations of variants and phenotypes. SKAT-O combined the burden test and SKAT to maximize the power [31]. Besides, by way of small-sample adjustment method of SKAT-O, we can properly control the type I error due to small sample size. However, SKAT-O also has its limitation: in the scenario that there are more neutral variants than actual deleterious variants, it may be slightly less powerful [32].

Our study suggests that variants in the *COL6A3* gene may increase susceptibility to PD. Further studies on the function and mechanism of *COL6A3* and other dystonia-related genes are needed to unravel the complexity of the association between PD and dystonia.

There are several limitations in our study. Our cohort only had a small sample size and mainly consisted of patients and controls from southeast China of Han Chinese populations. Therefore, the current study may produce false-positive results, or over-estimate the magnitude of the association, and the result might be population specific. Besides, population stratification might still exist even our participants nearly comes from the same region, leading to false-positive or false-negative findings. Therefore, future studies adjusting for population stratification via means of principal component analysis or multi-dimensional scaling are needed. Genetic relatedness might as well affect our conclusion since participant from both case or control may share alleles due to kinship rather than different PD condition. Methods such as kinship coefficients or identity by descent (IBD) might further be implemented to count for this potential bias. Currently, there are few studies of the relationship between *COL6A3* and PD. Further clinical studies with a larger sample size more and diverse demographic background are warranted, along with basic studies exploring the biological function of *COL6A3* and its associated pathways.

## Data Availability

The datasets used and/or analyzed during the current study are available from the corresponding author on reasonable request.
